# Rosai-Dorfman disease with renal involvement and associated autoimmune haemolytic anaemia in a 12-year-old girl: A case report

**DOI:** 10.1186/s12887-020-02368-3

**Published:** 2020-10-08

**Authors:** Tharmini Danisious, Mathula Hettiarachchi, Chanuka Dharmadasa, Heshan Jayaweera

**Affiliations:** 1 Professorial Paediatric Unit, Teaching Hospital, Peradeniya, Sri Lanka; 2 Sirimavo Bandaranaike Specialized Children’s Hospital, Peradeniya, Sri Lanka

**Keywords:** Renal Rosai-Dorfman, Sinus histiocytosis, Autoimmune haemolytic anaemia, Child, Case report

## Abstract

**Background:**

Rosai- Dorfman Disease (RDD) is a benign condition of unknown aetiology which is characterized by non-neoplastic proliferation of histiocytes. Pathophysiology and natural history remain obscure due to the low prevalence of disease. It is known to present with nodal or extranodal involvement and occurrence in the genitourinary system could lead to dreadful complications. RDD is diagnosed by demonstrating emperipolesis on histology and supported by S100 positivity in immunohistochemistry. Treatment is tailored individually and includes expectant monitoring, steroids, surgery, chemotherapy and radiotherapy. Prognosis will be poor if there is involvement of vital organs. We report a rare case of renal Rosai-Dorfman Disease in a 12-year-old girl which also associated with cold type autoimmune haemolytic anaemia (AIHA).

**Case presentation:**

A previously healthy, 12-year-old girl presented with low grade fever and cough over one month. On examination, she was pale, mildly icteric and had a firm mass in the left hypochondrial region.

Her blood count revealed significant eosinophilia, normocytic normochromic anaemia and thrombocytosis. Further laboratory investigations revealed reticulocytosis, positive urine urobilinogen, positive direct antiglobulin test and red blood cell agglutination on blood picture suggestive of autoimmune haemolytic anaemia.

Ultrasound scan of abdomen revealed paraaortic and left side retroperitoneal lymphadenopathy with left renal mass. It was further evaluated by Contrast Enhanced Computed Tomography (CECT). Biopsy was done and that concluded sinus histiocytosis with massive lymphadenopathy (SHML) with positive S100 and CD1a in immunohistochemistry. Child was treated with steroids however there was no significant response as assessed by repeat CT and has been commenced on chemotherapy.

**Conclusion:**

RDD is believed to be due to host immune dysregulation and precise diagnosis is imperative. It should be considered as differential diagnosis in a child presenting with massive lymphadenopathy and AIHA. Association between RDD and AIHA may possibly be explained by abnormal immune response of the host.

## Background

Sinus histiocytosis with massive lymphadenopathy (SHML), also known as Rosai- Dorfman Disease (RDD) is a benign self-limiting condition of unknown etiology which is characterized by non-neoplastic proliferation of histiocytes in the sinusoids of lymph nodes and in extra nodal tissues [1]. It has a prevalence of 1 in 200,000 [2]. It commonly occurs in the second and third decades of life [3, 4]. Pathophysiology and natural history remain concealed due to the low prevalence of disease. Postulated aetiologies include an exacerbated immune response to infections such as Epstein- Barr virus (EBV), Cytomegalovirus (CMV), Brucella, Klebsiella [5], human herpesvirus-6 (HHV-6) [6], or an autoimmune disease or a neoplastic process [7–10].

Diagnosis is established by demonstrating emperipolesis in histology and S100 positivity in immunohistochemistry [10–12]. Although no standard treatment has been established in children, it is tailored depending on the clinical presentation and involvement of vital organs. Expectant monitoring, steroids, surgery, chemotherapy and radiotherapy have been attempted with variable clinical response. We present a rare case of renal Rosai-Dorfman Disease associated with cold type autoimmune haemolytic anaemia (AIHA) which has not been previously described in the paediatric population.

## Case presentation

A previously healthy, 12-year-old girl presented with low grade fever of one-month duration associated with a non-productive cough. She did not have night sweats. On examination, she was afebrile, pale and icteric without significant lymphadenopathy. Her abdominal examination revealed a firm mass on the left hypochondriac region. Other system examinations including musculoskeletal and neurological examination were unremarkable.

Her initial blood count revealed normal white cell count with significant eosinophilia (11%), normocytic normochromic anaemia (6.3 g/dl) and thrombocytosis (533 × 10^3/uL). Further investigations revealed reticulocytosis (6.58%), positive urine urobilinogen and positive Direct Antiglobulin Test (DAT). Detailed DAT profile demonstrated negative IgG and positive C3d. Blood picture showed red blood cell agglutination, reticulocytosis with polychromasia and occasional spherocytes. With these findings, it was concluded as a cold type autoimmune haemolytic anaemia which was supported by elevated lactate dehydrogenase (LDH) level [LDH- 450 U/L (120–330 U/L)]. She had elevated total (27 μmol/L) and direct (17 μmol/L) bilirubin levels, but the gamma-glutamyl transferase (GGT) and alkaline phosphatase (ALP) were within the normal range.

Serum ferritin (430 ng/mL), erythrocyte sedimentation rate (125 mm/1st hour) and C-reactive protein (234 mg/l) were elevated. Renal function tests and rest of the liver profile remained normal throughout the course of illness.

**Fig. 1:**
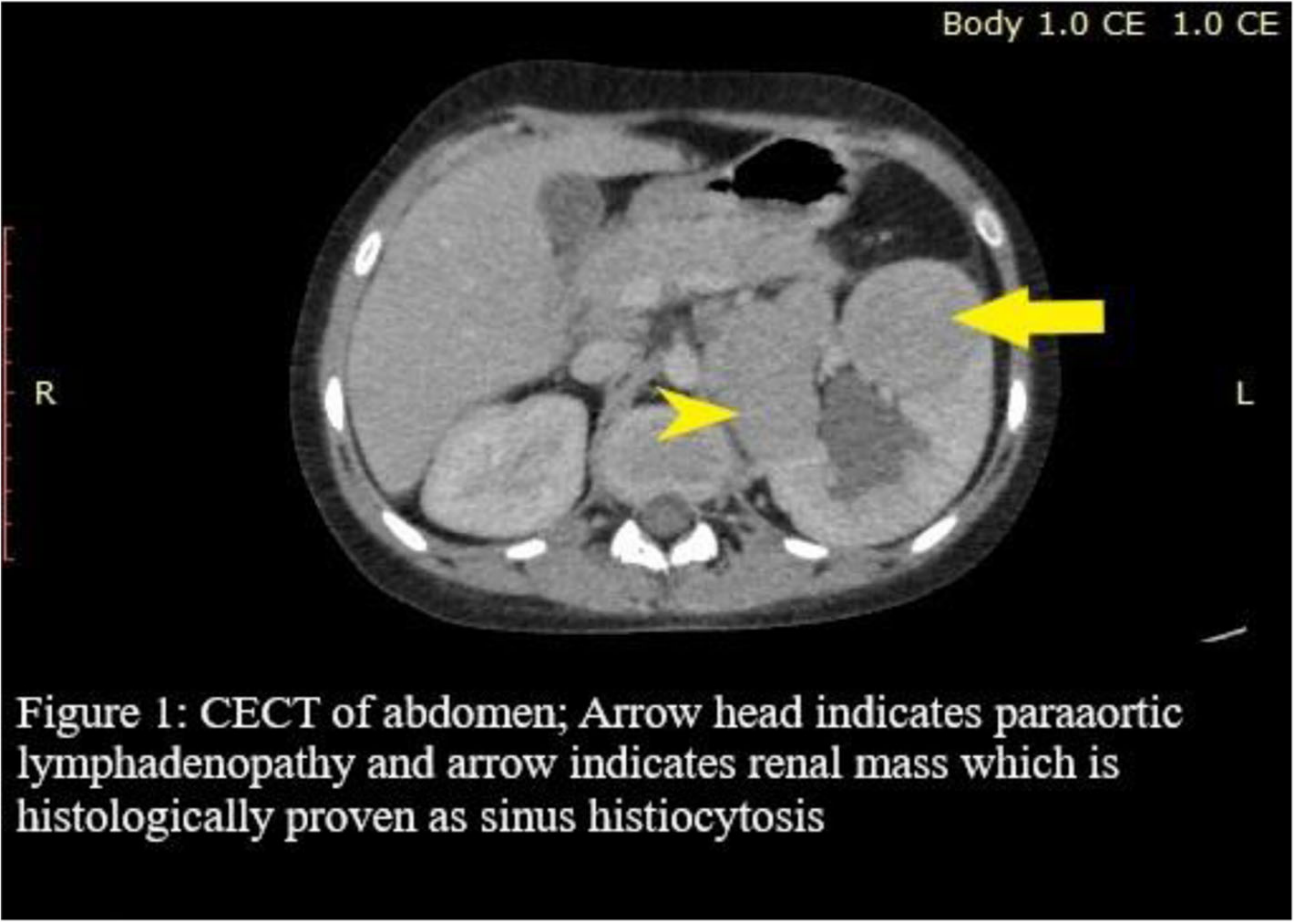
CECT of abdomen; Arrowhead indicates paraaortic lymphadenopathy and arrow indicates renal mass which is histologically proven as sinus histiocytosis

Concurrently, Ultrasound (US) scan of abdomen revealed a solitary mass confined to the left kidney with para aortic lymphadenopathy causing left ureteric compression and hydronephrosis. She was further investigated with CECT of chest and abdomen which concluded the same findings as in US scan [Fig. 1]. Primary tumor markers such as Alpha-fetoprotein (AFP) and Beta human chorionic gonadotropin (ß-hCG) were normal.

**Fig. 2:**
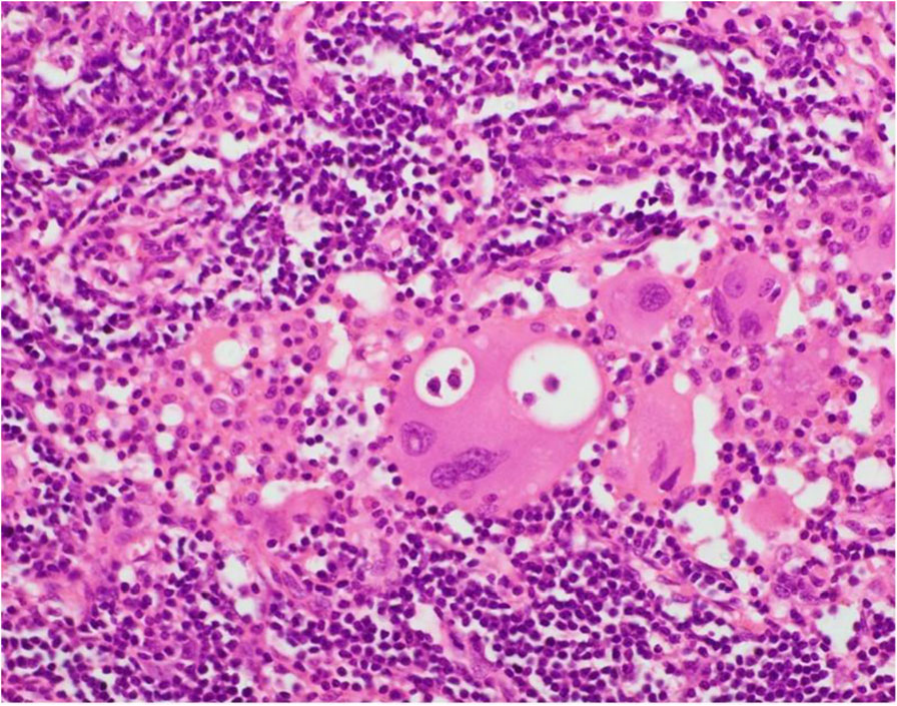
Histiocytes demonstrating emperipolesis within a left side perihilar lymph node (H&E × 400)

Suspecting possibility of haematological malignancy, bone marrow aspiration and trephine biopsy were undertaken and that too did not reveal any abnormal cells or evidence of bone marrow infiltration. US guided tru-cut biopsy of the retroperitoneal lymph node showed epithelioid looking cells in sinuses with reactive background of lymphoid tissue which was indecisive. Therefore, excisional biopsy of perihilar lymph node and a tru-cut biopsy from the renal mass were performed. The features of both biopsies were compatible with SHML with S100, CD68 and CD1a positivity in immunohistochemistry [Fig. 2].

Serum protein electrophoresis showed a polyclonal increase in gamma globulin without detection of any abnormal proteins. Her supplementary investigations showed hyperimmunoglobulinemia [IgG 2581 mg/dl (726–1085), IgA 338 mg/dl (70–229), IgM 130 mg/dl (35–72)] and hypoalbuminemia (23.7 g/L). Viral serology for EBV and CMV were negative. Furthermore, Antinuclear antibodies (ANA) and rheumatoid factor (RF) were also negative.

She was treated with oral prednisolone for three months but there was no significant response as assessed by a repeat CT. Subsequently she was commenced on multi-drug therapy consisting of mercaptopurine, methotrexate and vinblastine, however, she had marginal response to the therapy. She has been on regular follow-up to assess the progression of disease. Further treatment and follow-up will depend on her response to treatment.

## Discussion and conclusions

### Discussion

This rare disease entity was first described in 1965 by Rosai and Dorfman, however it was recognized as a clinical and pathological entity only after four years of its description [8].

Symptoms and physical findings depend on the organ involved and are widely variable. Lymphadenopathy being the main clinical manifestation and frequently involves cervical and submandibular regions. It can also affect axillary, inguinal and mediastinal regions. RDD significantly involves extra-nodal tissue (43%) and may be associated with other forms of histiocytosis [1]. The common sites for extra nodal involvement are skin, upper respiratory tract and bone [7]. However, it can occur in the genitourinary system, lower respiratory tract, oral cavity and soft tissues. Among the patients with extra-nodal involvement, approximately 75% have disease in head and neck region [13]. Involvement of lower respiratory tract, kidney or liver indicates poor prognosis [7].

In the literature, a 7-year old boy with diagnosis of RDD found to have renal involvement manifested as membranoproliferative glomerulonephritis during follow-up. He was treated with methylprednisolone and mizoribine and achieved complete remission [14]. Another study based on a computerized case registry that evaluated 220 patients, revealed glomerulonephritis in three patients [15]. In our patient, histological diagnosis of sinus histiocytosis was made from the biopsy of renal mass and to the best of our knowledge this is the first case of renal RDD in a child.

Furthermore, our patient developed cold AIHA that warranted blood transfusion. Although haemolytic anemia is an infrequent complication, association of AIHA with RDD has been described in the past [18–20]. Etiology of RDD is unknown, yet it is considered to be due to immune dysregulation. Approximately 10% of patients with RDD had coexisting immunological disease such as AIHA and rheumatoid arthritis (15,18). Up to 2018, only 4 paediatric cases of RDD associated with AIHA have been reported in the literature (20).

The practical difficulty in diagnosis is to distinguish RDD from sinus hyperplasia (sinus histiocytosis) due to the similarity in morphology, albeit well-defined expression of S100 protein in immunohistochemistry strongly associated with Rosai-Dorfman histiocytes and negative in normal sinus histiocytes [16]. Furthermore, Langerhans cell histiocytosis (LCH) is another differential diagnosis, characterized by clonal proliferation with different morphology to RDD and positive for both S100 and CD1a. In contrast, RDD is a S100 positive, CD1a negative histiocytes with emperipolesis of lymphocytes and other haematopoietic cells [17]. Occasionally, it may masquerade as lymphoma [3, 11].

Having a self-limiting course, RDD demands treatment for cases with pressure effects on vital organs [21]. Treatment options include expectant monitoring, steroids, chemotherapy and surgical excision with or without adjuvant radiotherapy [9]. High dose steroids exhibit response by a decline in lymph node size only during treatment while the use of methotrexate and 6-mercaptopurine for complicated RDD show prolonged response subsequent to tailing off medications [22]. Place for rituximab has been described in a child who later manifested systemic lupus erythematosus (SLE) [23]. Prednisolone and vinblastine were trialed to treat RDD that co-occurred with LCH [24].

Surgery is shown to be effective in cases of isolated lymphadenopathy without vital organ involvement and cutaneous or subcutaneous involvement [25]. Nevertheless, surgery can be disapproving in the latter due to the recurrent nature of the disease. In our patient, para-aortic and retroperitoneal lymphadenopathy was complicated with left hydronephrosis which will perhaps not be amenable to surgery or will have high risk due to surgery. Radiotherapy has shown to be effective in cutaneous RDD [25].

Irrespective of treatment, the prognosis of the disease is guarded with the reported fatality rate of 5–10% [26]. Regular long-term follow-up is indicated, and it includes assessing the progression of the disease, screening for concurrent diseases such as Sjogren syndrome, antiphospholipid syndrome and non-Hodgkin lymphoma [23, 25] and assessing the response for treatment.

### Conclusion

RDD is believed to be due to host immune dysregulation and precise diagnosis is imperative as it can mimic malignant lymphoproliferative disorders in children. However, it is more challenging due to the rarity of the disease, absence of significant symptoms related to the disease, involvement of multiple sites and non-specific findings on images. It should be considered as differential diagnosis in a child presenting with massive lymphadenopathy and AIHA. Association between RDD and AIHA may possibly be explained by abnormal immune response of the host.

## Data Availability

The relevant data and materials are available at the hospital in case if needed. Corresponding author can be contacted to find out the details regarding this case. The permission should be also obtained from the director of the hospital for accessing the data.

## Abbreviations

AFP: Alpha-fetoprotein
AIHA: Autoimmune haemolytic anaemia
ALP: Alkaline phosphatase
ANA: Antinuclear antibodies
ß-hCG: Beta human chorionic gonadotropin
CECT: Contrast Enhanced Computed Tomography
CMV: Cytomegalo Virus
DAT: Direct Antiglobulin Test
EBV: Epstein-Barr Virus
GGT: Gamma-glutamyl transferase
HHV-6: Human herpesvirus-6
LCH: Langerhans cell histiocytosis
LDH: Lactate dehydrogenase
RDD: Rosai-Dorfman Disease
RF: Rheumatoid factor
SHML: Sinus histiocytosis with massive lymphadenopathy
SLE: Systemic lupus erythematosus
US: Ultrasound
